# Defining the Landscape of Educational Experiences in Transplant Infectious Diseases: A National Survey of Infectious Diseases Fellows in the United States

**DOI:** 10.1093/ofid/ofae473

**Published:** 2024-08-20

**Authors:** Varun K Phadke, Saman Nematollahi, Julie M Steinbrink, Rachel Bartash, Megan K Morales, Scott C Roberts, Monica I Ardura, Nicole M Theodoropoulos

**Affiliations:** Division of Infectious Diseases, Emory University School of Medicine, Atlanta, Georgia, USA; Division of Infectious Diseases, University of Arizona College of Medicine, Tucson, Arizona, USA; Division of Infectious Diseases, Duke University School of Medicine, Durham, North Carolina, USA; Division of Infectious Diseases, Montefiore Medical Center/Albert Einstein College of Medicine, Bronx, New York, USA; Division of Infectious Diseases, Virginia Commonwealth University School of Medicine, Richmond, Virginia, USA; Section of Infectious Diseases, Yale School of Medicine, New Haven, Connecticut, USA; Department of Pediatrics, The Ohio State University College of Medicine, Columbus, Ohio, USA; Department of Internal Medicine, UMass Chan Medical School, Worcester, Massachusetts, USA

**Keywords:** fellowship training, infectious diseases fellows, medical education, transplant curriculum, transplant infectious diseases

## Abstract

**Background:**

Transplant infectious diseases (TID) is a growing area of expertise within infectious diseases (ID), but TID training is not standardized. Previous surveys of fellows identified opportunities to improve TID education resources but did not explore didactic, clinical, and nonclinical experiences comprehensively.

**Methods:**

The American Society of Transplantation ID Community of Practice surveyed adult and pediatric fellows in US-based general ID or dedicated TID training programs to explore their didactic exposure, clinical experiences, and non–direct patient care activities in TID.

**Results:**

A total of 234 fellows initiated the survey, and 195 (83%) (190 general ID and 19 TID fellows, including 125 adult, 76 pediatric, and 8 combined adult-pediatric fellows) completed the entire survey. More than half of the fellows described receiving no formal curricular content on most foundational topics in transplant medicine. Almost all respondents (>90%) had some inpatient TID experience, but for >60% of fellows this was <12 weeks annually. Clinical exposure varied by fellow and patient type—in an average month rotating on an inpatient TID service, more than half of adult fellows had evaluated ≥10 kidney, liver, or hematopoietic stem cell transplant recipients but <10 heart, lung, pancreas, or intestinal recipients; pediatric fellows saw <10 of all patient types. Nearly half (46%) of general ID fellows had not spent any time in the dedicated TID clinic at their program. Few fellows had participated in protocol development, organ selection meetings, or donor evaluations.

**Conclusions:**

This survey highlights important gaps in TID training. Given the increasing need for TID specialists, updated curricula and educational resources are needed.

Infection is an important cause of disease and death among immunocompromised hosts, including solid organ transplant (SOT) and hematopoietic cell transplant (HCT) recipients [1]. Given the large and growing number of transplant procedures [2, 3] and prevalence of immunosuppressive drug use [4], there is an increasing need for infectious diseases (ID) specialists with focused expertise in the care of transplant candidates and recipients, and other similarly immunocompromised hosts, often referred to as transplant ID (TID).

In 2010, the American Society of Transplantation (AST) ID Community of Practice (IDCOP) published recommendations for curricula for adult TID fellowship training [5]; subsequently, in 2013, the AST IDCOP collaborated with the Pediatric Infectious Diseases Society (PIDS) and the International Pediatric Transplant Association to publish an analogous document for pediatric TID fellowships [6]. These curricula were structured around the Accreditation Council for Graduate Medical Education (ACGME) core competencies and included recommendations for clinical, laboratory, and research training. Several programs now offer 1–2-year positions for graduates of general ID fellowship programs to pursue focused training in TID [7]; in addition, many general ID fellowship programs offer TID-focused “tracks”—formal or informal—allowing fellows more clinical and/or research exposure to TID during their general ID training. These tracks and fellowships are designed to prepare graduates for careers in which a significant portion of their time will be spent caring for this unique patient population.

Importantly, TID is not currently a subspecialty pathway accredited by the ACGME or any other professional society. As such, TID training experiences are not standardized between institutions. To understand fellow experiences in TID training, the AST IDCOP previously conducted surveys of adult and pediatric ID fellows [8, 9] that explored their self-reported clinical, didactic, and mentorship experiences, with the goal of identifying opportunities for improvement in TID fellowship training and educational resource development. Both surveys found that trainees desired more clinical exposure to TID and centralized learning resources to supplement their fellowship experiences. These findings translated into AST IDCOP and PIDS-led initiatives, such as a living online repository of key clinical articles, TransplantID.net [10], as well as online modules and monthly virtual case conferences for pediatric TID [11, 12].

However, these surveys also had limitations—for example, clinical and didactic exposure to TID were assessed based primarily on fellow-reported “adequacy” or “self-competency” (not patient volumes or other metrics), and neither survey explored fellows’ exposure to clinical activities that do not involve direct patient care (for example, protocol development and donor evaluation). To address these gaps, the AST IDCOP Education Work Group developed an updated survey of adult and pediatric ID and TID fellows to define the landscape of educational experiences in TID. The survey had several aims: (1) to explore clinical and didactic experiences at fellows’ home institutions with more granularity to provide insight into specific curricular needs; (2) to understand pedagogical approaches and resources used and preferred by fellows to provide guidance for educational resource development; and (3) to understand how fellows are exposed to non–patient care aspects of TID practice. The overall goal was to help inform future curricular recommendations to better prepare fellowship graduates for independent practice and professional growth.

## METHODS

The AST IDCOP Education Workgroup designed a 62-question online anonymous survey (Supplementary Figure 1) to explore the educational experiences of adult and pediatric ID fellows in TID, including (1) didactics, (2) inpatient and outpatient clinical exposure, and (3) nonclinical and professional development activities. ID fellows were asked to self-identify as being in an adult, pediatric, or combined adult-pediatric program and as general ID or TID fellows (ie, in a dedicated fellowship after their general ID fellowship), and general ID fellows were further asked to identify whether they were in a TID-focused track (formal or informal) within their fellowship. The survey was piloted with ID fellows and faculty not involved in the survey design, and the AST IDCOP Executive Committee and PIDS TID Committee leadership both reviewed and approved the final survey.

The survey was administered through Qualtrics, and responses were collected from 24 May to 1 September 2022. The survey link was distributed via email to adult and pediatric ID/TID fellowship program directors obtained through the American Medical Association FREIDA Online website and by manual review of the AST IDCOP and individual fellowship program websites. Fellows could access the survey link only through communication from their program director. To maintain respondent confidentiality and anonymity, center-specific data were not obtained, and any potential identifiers were removed before data analysis. The survey was promoted on program director listservs through AST, the Infectious Diseases Society of America (IDSA), and PIDS, and it was advertised directly to fellows on social media and a variety of free open access medical education platforms. As an incentive for survey completion, respondents could enter a raffle to win a $50 Amazon gift card.

Responses were divided into adult, pediatric, and TID fellows. Respondents who had started their fellowship within the past 3 months were excluded from the analysis, as were respondents from non-US programs. Data for each question were summarized with descriptive statistics based on the total number of respondents by question (since not all questions were required), and relevant comparisons between groups were performed using χ^2^ analysis.

This was an online anonymous survey conducted on behalf of the AST IDCOP Education Work Group. No identifying information about survey participants, training programs, or transplant centers was collected. The survey was reviewed and approved by the AST IDCOP Executive Committee and PIDS TID Committee leadership.

## RESULTS

### Demographics

The survey was sent to 227 ID fellowship programs (163 adult and 64 pediatric); a total of 234 individuals initiated the survey, and 195 (83%) completed the entire survey. The respondents included 190 general ID and 19 TID fellows (additional details shown in Table 1); data from 6 respondents from non-US programs were excluded.

**Table 1.** Infectious Diseases Fellows by Fellowship Category, Year, and Track

Fellowship Category, Year, and Track	ID Fellows, No. (%)			
	Adult	Pediatric	Combined Adult-Pediatric	Total
General ID fellows	108 (56.8)	74 (38.9)	8 (4.2)	190 (100)
Year 1	50 (46.3)	24 (32.4)	2 (25)	76 (100)
Year 2	48 (44.4)	25 (33.8)	2 (25)	75 (100)
Year 3+	10 (9.3)	25 (33.8)	4 (50)	39 (100)
Non–TID track	83 (76.9)	69 (93.2)	7 (87.5)	159 (100)
TID track	25 (23.1)	5 (6.8)	1 (12.5)	31 (100)
TID fellows	17 (89.5)	2 (10.5)	0 (0)	19 (100)
All ID fellows	125 (59.8)	76 (36.4)	8 (3.8)	209 (100)

Abbreviations: ID, infectious diseases; TID, transplant ID.

Most respondents (78%) reported wanting some of their time after fellowship to be focused on TID activities, with responses varying by training track (Supplementary Figure 1). More adult ID fellows in a TID track were interested in pursuing a dedicated TID fellowship after their general ID fellowship, compared with adult fellows not in a TID track (48% vs 3.7%, respectively; *P* < .001). Of fellows who wanted any of their time to be focused on TID after fellowship, half (50%) developed their interest in TID during the general ID fellowship, and a quarter (27%) developed their interest during residency. Factors that led to an interest in TID were patient care experiences (59% of respondents), fellowship didactics (40%), and clinical mentors (35%).

### Didactic Transplant Experiences

**Figure 1.**
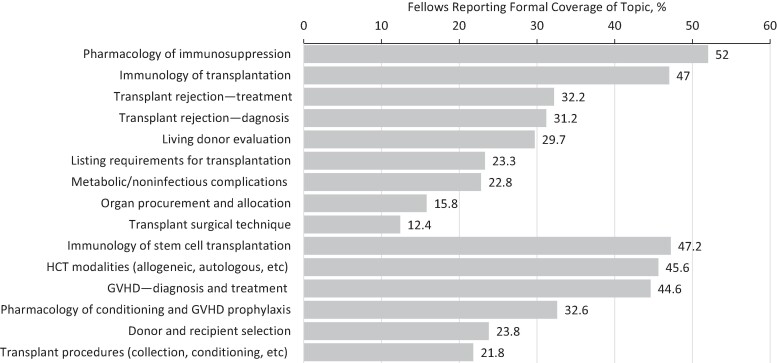
Didactic exposure to foundational topics in transplant medicine. Abbreviations: GVHD, graft-vs-host disease; HCT, hematopoietic cell transplant.

Less than half of all fellows—including TID fellows—reported formal exposure (defined as lecture, small group teaching, required self-paced module, or other didactic activity) to non–ID-specific topic areas pertinent to the general care of SOT and HCT recipients during their fellowship, with the only exception being pharmacology of SOT immunosuppression (52% said that this was formally covered) (Figure 1). Notably, 29% and 38% of general ID fellows reported that none of these foundational topics in SOT or HCT, respectively, were formally covered. When these topics were covered, the most common modalities that fellows identified were lectures (as part of an internal longitudinal curriculum) and case conferences. Fellows sought out a variety of external resources to learn about these foundational topics (Supplementary Table 1), with the 2 most used resources being review articles/textbooks and podcasts/social media (8.6%–22.5%, depending on topic) across all content areas. A minority of fellows (<10% for all content areas) reported using educational resources provided by AST to learn about SOT-specific topics.

### TID Inpatient Experiences

**Figure 2.**
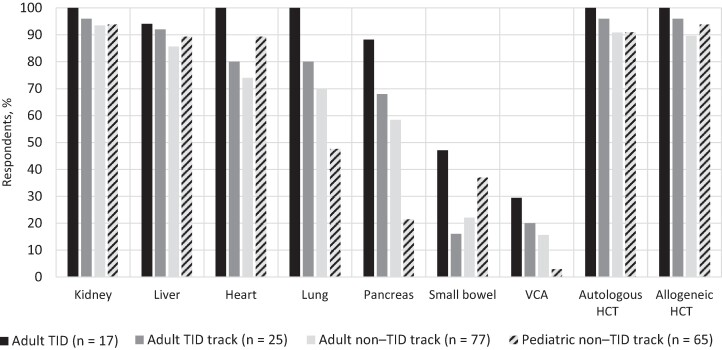
Types of transplants performed at survey respondent institutions. Abbreviations: HCT, hematopoietic cell transplant; TID, transplant infectious diseases; VCA, vascular composite allograft.

Most respondents indicated that their institutions performed kidney, liver, and heart transplants (94%, 87%, and 83% of respondents, respectively), as well as both autologous and allogeneic HCT (93% of respondents for both), whereas institutional experience with other types of transplants varied by fellow type (Figure 2).

Nearly all fellows (>90%) in all tracks reported having at least some inpatient exposure to TID, and >85% of these experiences were at the fellow's home institution. Most fellows (89% of 113 adult and 67% of 69 pediatric fellows) reported having a dedicated TID inpatient service at their institution. Two-thirds (n = 101 [66%]) of respondents (56% of adult ID and 85% of pediatric ID fellows) described this dedicated service as a “combined” service that sees both SOT and HCT recipients, and the remainder described separate consult services for SOT recipients and patients with HCT/hematologic cancer. Regardless of how their TID inpatient services were structured, or whether fellows identified as adult/pediatric or TID track or non–TID track, most general ID fellows (>60%) spent <12 weeks rotating on TID services each year; 19% of general ID fellows who described having a combined service spent <4 weeks per year rotating on that service.

**Figure 3.**
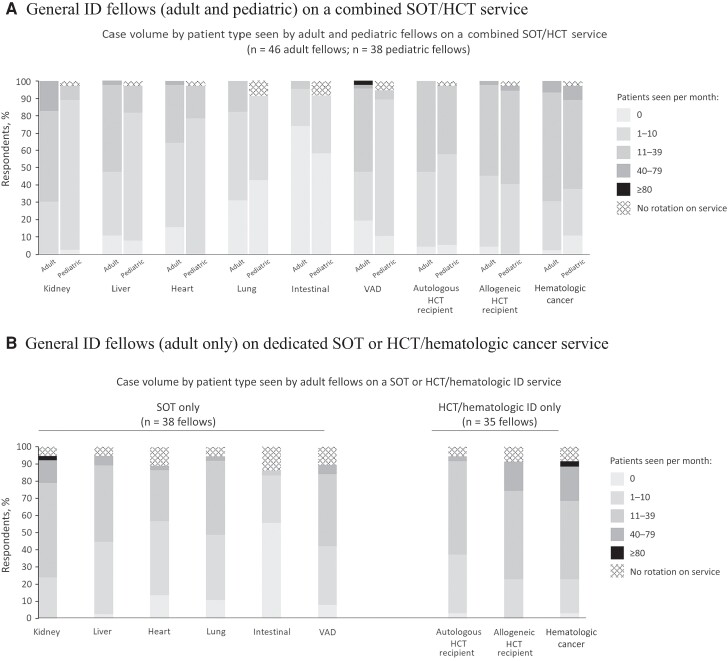
Fellows’ self-reported case volume by patient type on inpatient transplant infectious diseases (TID) consult services. *A,* General infectious disease (ID) fellows (adult and pediatric) on a combined solid organ transplant (SOT)/hematopoietic cell transplant (HCT) service. *B,* General ID fellows (adult only) on a dedicated SOT or HCT/hematologic cancer service. Abbreviation: VAD, ventricular assist device.

Clinical exposure (based on self-reported number of patients seen in an average month) varied by patient type and differed between adult and pediatric fellows. Limiting the analysis to general ID fellows rotating on a combined SOT/HCT TID service (the more common scenario), most adult fellows saw ≥10 of the following per month: kidney or liver transplant recipients, patients receiving ventricular assist device support, patients with hematologic cancer, or autologous/allogeneic HCT recipients (Figure 3*A*). In contrast, 65%, 82%, and 96% of adult fellows saw <10 heart, lung, or intestinal transplant recipients per month, respectively; findings were similar even among general ID fellows rotating on a SOT-focused service (Figure 3*B*). Among pediatric fellows, >75% of respondents reported seeing <10 of any SOT recipient or patients on ventricular assist device support in an average month; the only patient types that >50% of pediatric fellows reported seeing >10 cases per month were patients with hematologic cancer or allogeneic HCT recipients.

### TID Outpatient Experiences

**Figure 4.**
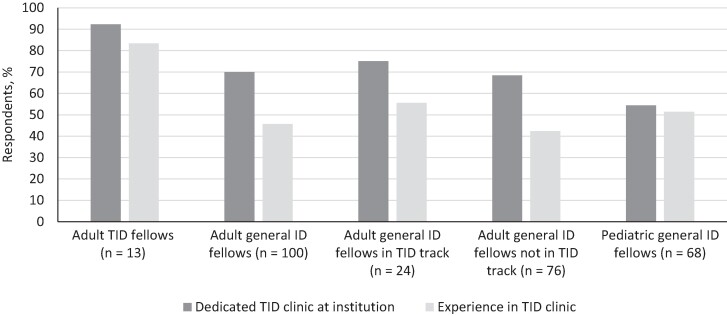
Experiences of transplant infectious diseases (TID) and general infectious diseases (ID) fellows in a dedicated TID/immunocompromised host clinic.

Outpatient clinical exposure was based on duration (number of TID clinic sessions) and case mix (self-reported number of patients seen in an average month rotating in the dedicated TID clinic). Two-thirds (n = 120 [66%]) of all respondents indicated that a dedicated TID clinic existed at their institution, but this varied by fellow type (Figure 4). Most (83%) of the adult TID fellows who reported having a dedicated TID clinic at their program had rotated there, compared with just 46% and 51% of adult and pediatric general ID fellows, respectively. Among general ID fellow respondents who had spent time in their institution's dedicated TID clinic (n = 32 adult fellows and n = 19 pediatric fellows), 65% experienced 1–4 half-day sessions per year (eg, once a week for ≤1 month); in contrast, 55% of TID fellows had experienced ≥25 half-day sessions per year (eg, at least once a week for >6 months).

Adult and pediatric fellows described different volumes and spectra of patient types during their TID clinic experiences (Supplementary Figure 3). For example, 39% of adult ID fellows reported seeing ≥5 kidney transplant recipients in an average month (or 4 half-day clinic sessions) rotating in a dedicated TID clinic; conversely, <5 of all other patient types were evaluated by most general adult ID fellows. Among pediatric ID fellows, >80% of respondents had evaluated <5 patients of any type in an average month.

### TID Nonclinical Experiences

Most general ID and TID fellows had never or only rarely participated in non–direct patient care activities that are relevant to TID practice after fellowship (Supplementary Figure 4), including attending relevant institutional meetings (eg, listing meetings and morbidity and mortality conferences), protocol development, or donor evaluations. The most common reasons cited by fellows for not participating in these activities were lack of awareness or activities not being offered to fellows.

### Professional Development in TID

Approximately one-third (n = 54 [29%]) of all fellows reported involvement in TID related research during fellowship (85% of TID fellows, 57% of general ID fellows in a TID track, and 19% of general ID fellows not in a TID track). Most fellows were able to disseminate their scholarly work, through a manuscript (40% of fellows), oral abstract presentation (21% of fellows), poster presentation (48% of fellows), or other venue (12% of fellows).

Among the survey participants who responded to questions about involvement in professional societies (n = 37), approximately two-thirds (n = 23 [62%]) were members of AST (100% of TID fellows, 82% of TID track fellows, and 29% of non–TID track general ID fellows who responded to this question); in contrast, only 3% and 5% were members of the International Society of Heart and Lung Transplantation or The Transplantation Society, respectively (and all of these fellow members were in a TID track or fellowship). Most fellows (87%) had not attended any transplant-specific professional society meetings during fellowship. The American Transplant Congress meeting had been attended by the largest number of fellows (n = 16), 75% of whom were in a TID track or fellowship.

## DISCUSSION

This AST IDCOP survey provides the first in-depth exploration of TID educational experiences among ID fellows in the United States. Our sample (190 general ID and 19 TID fellows; 74 pediatric fellows) was similar to or larger than previous surveys (203 general ID and 13 TID fellows in 2017 [8]; 60 pediatric fellows in 2014 [9]). Unlike previous surveys, the current survey subdivided general ID fellows into those in a self- or program-defined TID track; explored clinical and didactic experiences with more detail, including both inpatient and outpatient clinical experiences; and surveyed non–direct patient care experiences relevant to TID practice and professional development.

Several findings from this survey should inform TID curriculum and faculty development efforts. First, didactic and clinical exposure to transplant medicine and TID for most general ID fellows, including those self-identifying as being in a TID track, was limited in scope and depth, in both inpatient and outpatient settings. While fellows might have didactic exposure to TID-specific topics, many fellows had not been exposed formally to foundational topics in transplant medicine. These topics are relevant for all ID physicians, not only those who primarily practice TID, as many will be caring for the growing population of transplant recipients who go on to experience long-term complications, as well as other immunocompromised hosts [13].

Second, although most fellows reported at least some inpatient TID experience, this experience was short (<12 weeks per year for most and <4 weeks per year for many), with variable exposure to different patient types. Although it is difficult to define a minimum number of patients seen of each type as a training requirement, these data provide insight into the breadth and depth of supplemental educational content or adjunct clinical experiences that may be needed to ensure adequate exposure to these patient types during fellowship training. These needs are perhaps most acute for pediatric ID fellows, who reported less exposure to all patient types. Outpatient experiences in TID were similarly limited among both adult and pediatric fellows. This gap provides an opportunity to better prepare fellows for the longitudinal management of patients with immunocompromising conditions and associated infectious complications in the ambulatory setting, even when their future practice may or may not be affiliated with a transplant center.

Third, most ID fellows (including TID fellows) had limited experience in non–direct patient care activities that may be important components of their future role(s) in a transplant program, such as contributing to institutional protocol development, participating in donor evaluations, or attending organ selection or quality meetings. These activities are integral components of any transplant program and are an important, yet often unmeasured, contribution of TID to overall program success. Finally, many ID fellows reported not having the opportunity to engage with the wider transplant community, through membership or networking within transplant-specific professional societies. These findings suggest an opportunity for transplant professional societies to engage in more outreach and provide greater support for ID trainees; this could include facilitating attendance at fellow-specific meetings (eg, AST fellows meeting) or proactive recruitment of ID fellows into communities of practice that engage transplant professionals within and outside ID. In addition, programs and societies could more explicitly promote and incorporate nontraditional modalities for education and networking, such as social media and free open access medical education resources.

Other fellowships with a focus in transplant medicine have published accreditation requirements, curricula, and/or milestones to ensure and establish progressive professional competency. For example, the American College of Cardiology has published an advanced training statement for training in advanced heart failure and transplant cardiology [14], and the AST Transplant Nephrology Fellowship Training Accreditation Program certifies training programs in transplant nephrology [15]. Both transplant cardiology and transplant hepatology have specialty-specific milestones published by ACGME [16, 17]. These guidance documents are unified by specificity about curricular content, patient, and/or procedural volumes needed to develop proficiency and metrics to assess progressive knowledge and skill acquisition. Current recommendations for TID curricula are less explicit about patient volumes, case mix, or incremental levels of proficiency [5, 6]; although the results from this survey are skewed toward fellows with a preexisting interest in TID, our findings nevertheless provide some concrete metrics to inform updated recommendations to better reflect the evolving needs of ID trainees, and they provide guidance about the design and implementation of both TID tracks and fellowships.

Our findings are subject to several important limitations. First, the survey results may not be representative of all ID fellows, for a variety of reasons. The response rate among all ID fellows in the United States was low; based on data from the National Resident Matching Program, a total of 687 individuals matched in adult general ID fellowships in 2021 (n = 365) and 2020 (n = 322), and a total of 125 individuals matched in pediatric general ID fellowships in 2021 (n = 42), 2020 (n = 46), and 2019 (n = 37) [18]. Assuming 50% of second-year adult general ID fellows pursue additional years of training (and all pediatric general ID fellows complete 3 years of training), the estimated survey response rate among all US general ID fellows was 19.5% (12.7% for adult and 55.2% for pediatric fellows). While there are no match data for TID fellowships, there are <30 dedicated TID fellowships in the United States (including adult and pediatric programs), from which 19 respondents participated in the survey. In addition, not all respondents completed the entire survey, and these missing data could affect the overall conclusions. Finally, since most respondents expressed at least some interest in TID practice after fellowship, our findings may be skewed toward the perspective of trainees with a preexisting interest in TID and therefore may not represent the experience of all ID fellows or trainees in programs outside the United States.

Second, data from respondents to this survey may not reflect actual educational experiences of ID fellows. Clinical and didactic exposure were assessed based on self-report, which is subject to recall bias. Our survey was conducted in the wake of the coronavirus disease 2019 pandemic, which may have disrupted didactic, clinical, and other experiences for ID fellows during the survey period. Given that the survey was anonymous, and that we did not capture respondents’ institutions, we are unable to corroborate survey responses with publicly available information regarding fellowship curricular content or transplant center volumes. Similarly, we did not identify respondents by institution, so it is possible that multiple fellows from the same training program completed the survey or that a single individual could complete the survey more than once. However, since most ID fellowship programs are small (<3 fellows per year) and larger programs are more likely to be affiliated with an academic center and/or transplant program, responses from fellows in the same program would likely bias us toward overestimating TID educational experiences during fellowship training. Finally, only a small number of TID track and TID fellows were captured by the survey, and we did not define tracks formally, so we can draw limited conclusions about the details of these training pathways, which likely vary significantly between programs; a more in-depth exploration of these programs using targeted surveys and interviews of program directors and fellows may be helpful.

In conclusion, we identified several important gaps in TID training for general ID and TID fellows. As the population of immunocompromised hosts continues to grow, training in TID is relevant to all ID physicians. These training experiences require both curricular and faculty development and support to be sustainable and effective. Investing in the training of the next generation of TID physicians through evidence-based pedagogy, resources for self-directed learning, and experiential skill acquisition, as well as institutional development and support of TID clinicians and educators, stands to benefit all stakeholders, including learners, programs, and patients.

## Supplementary Data

Supplementary materials are available at *Open Forum Infectious Diseases* online. Consisting of data provided by the authors to benefit the reader, the posted materials are not copyedited and are the sole responsibility of the authors, so questions or comments should be addressed to the corresponding author.

## Supplementary Material

ofae473_Supplementary_Data
