# Supplementary material for: Defining the Landscape of Educational Experiences in Transplant Infectious Diseases: A National Survey of Infectious Diseases Fellows in the United States
Source: Open Forum Infect Dis. 2024 Aug 20;11(9):ofae473. doi: 10.1093/ofid/ofae473 (PMC11389608; doi:10.1093/ofid/ofae473)
Supplement: ofae473_Supplementary_Data [file ofae473_supplementary_data.zip › ast idcop fellows survey.pdf]

EMORY UNIVERSITY SCHOOL OF MEDICINE

Introduction

Welcome!

This survey is being conducted by the AST ID Community of Practice (COP) to characterize the clinical and educational experiences of ID fellows in the field of Transplant ID. As the need for ID providers with experience in Transplant ID has increased, the AST IDCOP is interested in updating its recommended curricula for training in TID - this is relevant whether or not you intend to pursue a career focused primarily on TID.

This survey is completely ANONYMOUS - you will not be required to identify yourself or your training program. We will ask you to create a unique identifier before beginning the survey. The survey should not take more than 10-15 minutes to complete.

If you wish to be entered into a drawing to win a $50 Amazon gift card, you may enter a personal email address at the end

of the survey.

We appreciate your time and are excited to share our results with you!

-Varun Phadke (Chair, Education Workgroup in the AST IDCOP)
-Julie Steinbrink (Co-Chair, Education Workgroup in the AST IDCOP)

Please create a unique identifier below by entering the first letter of your last name followed by the last 4 digits of your phone number (for example, for J. Smith with number 123-456-7890, the identifier would be S7890)

Demographics

In what year did you BEGIN your CURRENT fellowship training program?

- ○ 2022 (I JUST began fellowship)
- ○ 2021 (I am a FIRST year fellow)

- 2020 (I am a SECOND year fellow)
- 2019 (I am a THIRD year fellow)
- [ ] Other

Are you CURRENTLY in an adult or pediatric fellowship training program?

- Adult
- Pediatric
- Combined adult/pediatric

Are you CURRENTLY in a focused Transplant ID/Immunocompromised Host training program or pathway?

- No
- Yes - A dedicated fellowship AFTER my general ID fellowship
- Yes - A "track" within my general ID fellowship (formal or informal)

In what country is your CURRENT training program?

Are you interested in pursuing a dedicated Transplant ID/Immunocompromised Host fellowship AFTER your general ID fellowship?

- Yes
- No
- Maybe

Is your dedicated Transplant ID/Immunocompromised Host fellowship at the same institution as your general ID fellowship?

- Yes - Same institution
- No - Different institution

What is the focus of your dedicated Transplant ID/Immunocompromised Host fellowship?

- Solid organ transplantation
- Oncology and stem cell transplantation
- Both solid organ transplantation and oncology/HSCT ID

If it were an option available to you, would you consider pursuing additional dedicated training (AFTER your general ID

fellowship) specifically in pediatric transplant infectious diseases?

- Yes
- No
- Maybe

What proportion of your time after fellowship would you like to focus on activities related to Transplant ID?

- None
- Some, but less than half
- About half
- More than half, but not all
- All
- Not sure

When did you first become interested in Transplant ID?

- Before medical school
- Medical school
- Residency
- Fellowship
- I'm not interested in Transplant ID
- Other

How did you become interested in Transplant ID? (select all that apply)

- ☐ Research you have done in the field
- ☐ Personal health experience (personal, family, other)
- ☐ Clinical mentor
- ☐ Research mentor
- ☐ Classes in medical school
- ☐ Didactics in residency
- ☐ Didactics in ID fellowship
- ☐ Patient care experience
- ☐ Social media
- ☐ [ ] Other

Which of the following types of transplant are performed at YOUR institution? (select all that apply, even if only performed as part of a combined transplant, e.g., if your institution only performs simultaneous kidney-pancreas transplants and not isolated pancreas transplants, you should still select BOTH kidney and pancreas)

- ☐ Kidney
- ☐ Pancreas
- ☐ Liver

- ☐ Heart
- ☐ Lung
- ☐ Small bowel
- ☐ Vascular composite allograft (face, limb, larynx, etc.)
- ☐ Autologous stem cell
- ☐ Allogeneic stem cell
- ☐ None of the above

Educational Experiences in Transplant Medicine

The following are core topics in solid organ transplant (SOT) medicine that are not specific to infectious diseases.

Indicate which content areas are covered FORMALLY (lecture, small group teaching, required self-paced module, other didactic activity, etc.) during your CURRENT fellowship training program (select all that apply)

- ☐ Immunology of organ transplantation
- ☐ Pharmacology of immunosuppressive therapy
- ☐ Transplant surgical technique and complications
- ☐ Organ procurement and allocation
- ☐ Listing requirements for transplantation
- ☐ Living donor evaluation
- ☐ Transplant rejection - diagnosis
- ☐ Transplant rejection - treatment
- ☐ Metabolic and other non-infectious complications of organ transplantation

☐ None of the above were covered formally

Indicate the modalities that are used to cover this content about organ transplantation during your fellowship training (select all that apply)

	Part of an internal longitudinal curriculum (e.g., lecture)	Journal club (internal)	Case conference (internal)	Grand rounds (internal)
» Immunology of organ transplantation	☐	☐	☐	☐
» Pharmacology of immunosuppressive therapy	☐	☐	☐	☐
» Transplant surgical technique and complications	☐	☐	☐	☐
» Organ procurement and allocation	☐	☐	☐	☐
» Listing requirements for transplantation	☐	☐	☐	☐
» Living donor evaluation	☐	☐	☐	☐
» Transplant rejection - diagnosis	☐	☐	☐	☐
» Transplant rejection - treatment	☐	☐	☐	☐

	Part of an internal longitudinal curriculum (e.g., lecture)	Journal club (internal)	Case conference (internal)	Grand rounds (internal)
» Metabolic and other non-infectious complications of organ transplantation	☐	☐	☐	☐
» None of the above were covered formally	☐	☐	☐	☐

Which of the following external resources, if any, have you used to learn this content about organ transplantation during your fellowship training? (select all that apply)

	Review articles/textbooks	Podcast or social media	Self-paced interactive online module	AST webinar	AST Comprehensive Trainee Curriculum	AST Symp Transp
Immunology of organ transplantation	☐	☐	☐	☐	☐	
Pharmacology of immunosuppressive therapy	☐	☐	☐	☐	☐	
Transplant surgical technique and complications	☐	☐	☐	☐	☐	
Organ procurement and allocation	☐	☐	☐	☐	☐	

	Review articles/textbooks	Podcast or social media	Self-paced interactive online module	AST webinar	AST Comprehensive Trainee Curriculum	AST Symp Transp
Listing requirements for transplantation	☐	☐	☐	☐	☐	
Living donor evaluation	☐	☐	☐	☐	☐	
Transplant rejection - diagnosis	☐	☐	☐	☐	☐	
Transplant rejection - treatment	☐	☐	☐	☐	☐	
Metabolic and other non-infectious complications of organ transplantation	☐	☐	☐	☐	☐	

What other resource(s) have you used to learn about immunology of transplantation?

What other resource(s) have you used to learn about pharmacology of immunosuppressive therapy?

What other resource(s) have you used to learn about transplant surgical technique and complications?

What other resource(s) have you used to learn about organ procurement and allocation?

What other resource(s) have you used to learn about listing requirements for transplantation?

What other resource(s) have you used to learn about living donor evaluation?

What other resource(s) have you used to learn about transplant rejection diagnosis?

What other resource(s) have you used to learn about transplant rejection treatment?

What other resource(s) have you used to learn about metabolic and other non-infectious complications of organ transplantation?

The following are core topics in hematopoietic stem cell transplant (HSCT) that are not specific to infectious diseases.

Indicate which content areas are covered FORMALLY (lecture, small group teaching, required self-paced module, other didactic activity, etc.) during your CURRENT fellowship training program (select all that apply)

- ☐ Immunology of stem cell transplantation (match, timeline of immune reconstitution, etc.)
- ☐ Donor and recipient selection
- ☐ HSCT modalities (allogeneic/autologous/cord blood, related/unrelated, etc.)
- ☐ Transplant procedures (stem cell collection, conditioning, engraftment, etc.)
- ☐ Graft versus host disease - diagnostics and therapy
- ☐ Pharmacology of conditioning and GVHD prophylaxis regimens
- ☐ None of the above were covered formally

Indicate the modalities that are used to cover this content about hematopoietic transplantation during your fellowship training (select all that apply)

	Part of an internal longitudinal curriculum (e.g., lecture)	Journal club (internal)	Case conference (internal)	Grand Rounds (internal)
» Immunology of stem cell transplantation (match, timeline of immune reconstitution, etc.)	☐	☐	☐	☐
» Donor and recipient selection	☐	☐	☐	☐

	Part of an internal longitudinal curriculum (e.g., lecture)	Journal club (internal)	Case conference (internal)	Grand Rounds (internal)
» HSCT modalities (allogeneic/autologous/cord blood, related/unrelated, etc.)	☐	☐	☐	☐
» Transplant procedures (stem cell collection, conditioning, engraftment, etc.)	☐	☐	☐	☐
» Graft versus host disease - diagnostics and therapy	☐	☐	☐	☐
» Pharmacology of conditioning and GVHD prophylaxis regimens	☐	☐	☐	☐
» None of the above were covered formally	☐	☐	☐	☐

Which of the following external resources, if any, have you used to learn this content about hematopoietic transplantation during your fellowship training? (select all that apply)

	Review articles/textbooks	Podcast or social media	Self-paced interactive online module	Webinar	Other resource not listed	I did use exte reso to le th top
Immunology of stem cell transplantation (match, timeline of immune reconstitution, etc.)	☐	☐	☐	☐	☐	☐
Donor and recipient selection	☐	☐	☐	☐	☐	☐
HSCT modalities (allogeneic/autologous/cord blood, related/unrelated, etc.)	☐	☐	☐	☐	☐	☐
Transplant procedures (stem cell collection, conditioning, engraftment, etc.)	☐	☐	☐	☐	☐	☐
Graft versus host disease - diagnostics and therapy	☐	☐	☐	☐	☐	☐
Pharmacology of conditioning and GVHD prophylaxis regimens	☐	☐	☐	☐	☐	☐

What other resource(s) have you used to learn about immunology of stem cell transplantation?

What other resource(s) have you used to learn about donor and recipient selection for stem cell transplantation?

What other resource(s) have you used to learn about HSCT modalities?

What other resource(s) have you used to learn about stem cell transplant procedures (stem cell collection, conditioning, engraftment, etc.)?

What other resource(s) have you used to learn about graft versus host disease diagnostics and therapy?

What other resource(s) have you used to learn about pharmacology of conditioning and GVHD prophylaxis regimens?

Inpatient Clinical Experiences in Transplant ID

During your CURRENT fellowship, have you had any inpatient Transplant ID experience (on either a dedicated Transplant ID/Immunocompromised Host service or a General ID service)?

- ○ Yes
- ○ No

On what type of inpatient service was your inpatient Transplant ID experience? (select all that apply)

- ☐ A dedicated Transplant ID/Immunocompromised Host service

- [ ] A General ID service

Was your experience on a dedicated Transplant ID/Immunocompromised Host service at your own institution or a different institution (away rotation)?

- ○ My institution
- ○ Different institution (away rotation)
- ○ Both

Does your institution have a dedicated Transplant ID/Immunocompromised Host inpatient consult service (or services)?

- ○ Yes
- ○ No

How are patients divided on the dedicated service(s)?

- ○ Separate services for SOT and HSCT/heme malignancy (i.e. different consult teams see either SOT or HSCT/heme malignancy patients)
- ○ Combined immunocompromised host service (i.e. the same consult team(s) sees both SOT and HSCT/heme malignancy patients)

On average, how many WEEKS PER YEAR do fellows in your current program spend on the dedicated SOT ID inpatient service? (assume that 1 month = 4 weeks)

- None
- 1 to 4 weeks
- >4 to 12 weeks
- >12 to 24 weeks
- >24 weeks

In an average month (4 weeks) on the SOT ID inpatient service, how many of the following patient types do you evaluate?

	0	<10 (~1-2 per week)	10 to <40 (~3-10 per week)	40 to <80 (10-20 per week)	>80 (more than 20 per week)	I have not rotated on this service
Kidney transplant recipient	○	○	○	○	○	○
Liver transplant recipient	○	○	○	○	○	○
Heart transplant recipient	○	○	○	○	○	○
Lung transplant recipient	○	○	○	○	○	○
Intestinal transplant recipient	○	○	○	○	○	○

	0	<10 (~1-2 per week)	10 to <40 (~3-10 per week)	40 to <80 (10-20 per week)	>80 (more than 20 per week)	I have not rotated on this service
Multi-visceral transplant recipient	○	○	○	○	○	○
Patient with ventricular assist device (VAD)	○	○	○	○	○	○

On average, how many WEEKS PER YEAR do fellows in your current program spend on the dedicated HSCT/heme malignancy ID inpatient service? (assume that 1 month = 4 weeks)

- ○ None
- ○ 1 to 4 weeks
- ○ >4 to 12 weeks
- ○ >12 to 24 weeks
- ○ >24 weeks

In an average month (4 weeks) on the HSCT/heme malignancy ID inpatient service, how many of the following patient types do you evaluate?

	0	<10 (~1-2 per week)	10 to <40 (~3-10 per week)	40 to <80 (10-20 per week)	>80 (more than 20 per week)	I have not rotated on this service
Recipient of autologous SCT	○	○	○	○	○	○
Recipient of allogeneic SCT	○	○	○	○	○	○
Patient with hematologic malignancy (not SCT recipient)	○	○	○	○	○	○
Patient with primary immunodeficiency	○	○	○	○	○	○

On average, how many WEEKS PER YEAR do fellows in your current program spend on the dedicated Transplant ID/Immunocompromised Host inpatient service? (assume that 1 month = 4 weeks)

- ○ None
- ○ 1 to 4 weeks
- ○ >4 to 12 weeks
- ○ >12 to 24 weeks
- ○ >24 weeks

In an average month (4 weeks) on the Transplant ID/Immunocompromised Host inpatient service, how many of

the following patient types do you evaluate?

	0	<10 (~1-2 per week)	10 to <40 (~3-10 per week)	40 to <80 (10-20 per week)	>80 (more than 20 per week)	I have not rotated on this service
Kidney transplant recipient	○	○	○	○	○	○
Liver transplant recipient	○	○	○	○	○	○
Heart transplant recipient	○	○	○	○	○	○
Lung transplant recipient	○	○	○	○	○	○
Intestinal transplant recipient	○	○	○	○	○	○
Multi-visceral transplant recipient	○	○	○	○	○	○
Patient with ventricular assist device (VAD)	○	○	○	○	○	○
Recipient of autologous SCT	○	○	○	○	○	○
Recipient of allogeneic SCT	○	○	○	○	○	○
Patient with hematologic malignancy (not SCT recipient)	○	○	○	○	○	○
Patient with primary immunodeficiency	○	○	○	○	○	○

In an average month (4 weeks) on a GENERAL ID inpatient service, how many of the following patient types do you evaluate?

	0	<10 (~1-2 per week)	10 to <40 (~3-10 per week)	40 to <80 (10-20 per week)	>80 (more than 20 per week)
Kidney transplant recipient	○	○	○	○	○
Liver transplant recipient	○	○	○	○	○
Heart transplant recipient	○	○	○	○	○
Lung transplant recipient	○	○	○	○	○
Intestinal transplant recipient	○	○	○	○	○
Multi-visceral transplant recipient	○	○	○	○	○
Patient with ventricular assist device (VAD)	○	○	○	○	○
Recipient of autologous SCT	○	○	○	○	○
Recipient of allogeneic SCT	○	○	○	○	○
Patient with hematologic malignancy (not SCT recipient)	○	○	○	○	○

	0	<10 (~1-2 per week)	10 to <40 (~3-10 per week)	40 to <80 (10-20 per week)	>80 (more than 20 per week)
Patient with primary immunodeficiency	○	○	○	○	○

Outpatient Clinical Experiences in Transplant ID

Does your program have a dedicated Transplant ID/Immunocompromised Host clinic?

○ Yes
○ No

During your CURRENT fellowship, have you had an outpatient transplant ID experience? (select all that apply)

☐ Yes - In a dedicated Transplant ID/Immunocompromised Host clinic
☐ Yes - Transplant patients (SOT or HSCT) seen in a general ID clinic
☐ No

On average, how many clinic sessions per YOUR CURRENT YEAR of training are spent in a dedicated Transplant ID/Immunocompromised Host clinic?

- ○ 1 to 4 sessions (for example, once a week for a month or fewer)
- ○ 5 to 12 sessions (for example, once a month)
- ○ 13 to 24 sessions (for example, once a week for 3-6 months)
- ○ 25 to 48 sessions (for example, once a week for >6-12 months)
- ○ >48 sessions (for example, more than once a week)

In an average month (or 4 half-day clinic sessions) in a dedicated Transplant ID/Immunocompromised Host clinic, how many of the following patient types do you evaluate?

	0	<5 (at most 1 per session)	5 to 10 (1-2 per session)	>10 (more than 2 per session)
Kidney transplant recipient	○	○	○	○
Liver transplant recipient	○	○	○	○
Heart transplant recipient	○	○	○	○
Lung transplant recipient	○	○	○	○
Intestinal transplant recipient	○	○	○	○
Multi-visceral transplant recipient	○	○	○	○
Patient with ventricular assist device (VAD)	○	○	○	○
Recipient of autologous SCT	○	○	○	○

	0	<5 (at most 1 per session)	5 to 10 (1-2 per session)	>10 (more than 2 per session)
Recipient of allogeneic SCT	○	○	○	○
Patient with hematologic malignancy (not SCT recipient)	○	○	○	○
Patient with primary immunodeficiency	○	○	○	○

On average, how many clinic sessions per YOUR CURRENT YEAR of training are spent in a General ID clinic?

- ○ 1 to 4 sessions (for example, once a week for a month or fewer)
- ○ 5 to 12 sessions (for example, once a month)
- ○ 13 to 24 sessions (for example, once a week for 3-6 months)
- ○ 25 to 48 sessions (for example, once a week for >6-12 months)
- ○ >48 sessions (for example, more than once a week)

In an average month (or 4 half-day clinic sessions) in general ID clinic, how many of the following patient types do you evaluate?

	0	<5 (at most 1 per session)	5 to 10 (1-2 per session)	>10 (more than 2 per session)
Kidney transplant recipient	○	○	○	○

	0	<5 (at most 1 per session)	5 to 10 (1-2 per session)	>10 (more than 2 per session)
Liver transplant recipient	○	○	○	○
Heart transplant recipient	○	○	○	○
Lung transplant recipient	○	○	○	○
Intestinal transplant recipient	○	○	○	○
Multi-visceral transplant recipient	○	○	○	○
Patient with ventricular assist device (VAD)	○	○	○	○
Recipient of autologous SCT	○	○	○	○
Recipient of allogeneic SCT	○	○	○	○
Patient with hematologic malignancy (not SCT recipient)	○	○	○	○
Patient with primary immunodeficiency	○	○	○	○

Non-Clinical Experiences in Transplant ID

During your CURRENT fellowship, how often have you attended/participated in the following activities?

	Never	Rarely	Sometimes	Frequently	Always
Organ listing meetings	○	○	○	○	○
Organ quality meetings	○	○	○	○	○
Donor evaluation	○	○	○	○	○
Protocol development	○	○	○	○	○
Organ procurement organization (OPO) meetings	○	○	○	○	○
Transplant M&M/QAPI (Quality Assurance and Performance Improvement) meetings	○	○	○	○	○

For each of these activities in which you did not participate or participated only rarely, what was the reason?

	Not aware of this activity	Not offered to fellows	Not enough time because of clinical/research/other duties	Not interested	No such activity at my program
Organ listing meetings	○	○	○	○	○
Organ quality meetings	○	○	○	○	○
Donor evaluation	○	○	○	○	○
Protocol development	○	○	○	○	○

	Not aware of this activity	Not offered to fellows	Not enough time because of clinical/research/other duties	Not interested	No such activity at my program
Organ procurement organization (OPO) meetings	○	○	○	○	○
Transplant M&M/QAPI (Quality Assurance and Performance Improvement) meetings	○	○	○	○	○

Research Experiences in Transplant ID

During your current fellowship, were you involved in transplant ID related research?

- ○ Yes
- ○ No

What type(s) of research? (select all that apply)

- ☐ Basic science
- ☐ Clinical/Translational
- ☐ Quality Improvement
- ☐ Epidemiology
- ☐ Medical Education

☐ [ ] Other

Were you able to disseminate your scholarly work during your transplant ID fellowship or track? (select all that apply)

☐ Yes - manuscript
☐ Yes - oral abstract presentation
☐ Yes - poster presentation
☐ [ ] Yes - other
☐ No

Was there a research requirement as part of your Transplant ID/Immunocompromised Host fellowship?

○ Yes
○ No

What was the requirement?

[ ]

Have you attended the AST Fellows Symposium on Transplantation?

- Yes
- No

Why not? (select all that apply)

- Not aware of this meeting
- Lack of protected time
- Lack of funds
- Not interested in attending
- Other

Which of the following transplant specific meetings have you attending during your fellowship training? (select all that apply)

- American Transplant Congress (ATC)
- International Society of Heart and Lung Transplantation (ISHLT) Annual Meeting
- The Transplantation Society (TTS)
- Cutting Edge of Transplantation (CEOT)
- Other

- [ ] None of the above

Are you a member of any of the following organizations?

- [ ] American Society of Transplantation (AST)
- [ ] International Society of Heart and Lung Transplantation (ISHLT)
- [ ] The Transplantation Society (TTS)
- [ ] ____ Other

COVID-19 and Transplant ID Education

Which aspects of your training experience have been impacted by the COVID-19 pandemic? (select all that apply)

- [ ] Educational conferences
- [ ] Clinical experience
- [ ] Clinical volume
- [ ] Research projects
- [ ] ____ Other
- [ ] None of the above

Please describe the changes you experienced in these aspects of your training

Drawing

Thank you for taking the time to complete this survey!

Would you like to be entered into a drawing to win a $50 Amazon gift card? Your responses will still remain anonymous.

- Yes
- No

Powered by Qualtrics
