# Supplementary material for: Defining the Landscape of Educational Experiences in Transplant Infectious Diseases: A National Survey of Infectious Diseases Fellows in the United States
Source: Open Forum Infect Dis. 2024 Aug 20;11(9):ofae473. doi: 10.1093/ofid/ofae473 (PMC11389608; doi:10.1093/ofid/ofae473)
Supplement: ofae473_Supplementary_Data [file ofae473_supplementary_data.zip › Supplemental Figures and table.docx]

**Supplemental Figures**

**Supplemental Figure 1: Survey**

**Supplemental Figure 2: Career Interests of Survey Respondents**

Abbreviations: ID, Infectious Diseases; TID, Transplant Infectious Diseases

**Supplemental Table 1: Percent of Fellows Utilizing External Resources to Learn Foundational Topics in Transplant Medicine**

|  | Review article/ textbook | Podcast/ social media | Case-based module | AST CTC | AST Webinar |
| --- | --- | --- | --- | --- | --- |
| *Topic in SOT* | | | | | |
| Pharmacology of immunosuppression | 74% | 19% | 5% | 9% | 7% |
| Immunology of SOT | 73% | 23% | 9% | 9% | 6% |
| Treatment of rejection | 64% | 13% | 6% | 8% | 4% |
| Diagnosis of rejection | 63% | 14% | 7% | 7% | 3% |
| Living donor evaluation | 50% | 15% | 9% | 6% | 3% |
| Listing requirements for transplant | 44% | 11% | 7% | 7% | 4% |
| Metabolic and other non-infectious complications of SOT | 52% | 12% | 5% | 6% | 3% |
| Organ procurement and allocation | 38% | 11% | 6% | 7% | 5% |
| Transplant surgical technique | 40% | 9% | 6% | 6% | 2% |
| *Topic in HCT* | | | | | |
| Immunology of HCT | 73% | 15% | 6% |  |  |
| HCT modalities (autologous, allogeneic, etc.) | 70% | 12% | 5% |  |  |
| Donor and recipient selection | 51% | 11% | 6% |  |  |
| Transplant procedures (cell collection, conditioning, etc.) | 59% | 9% | 6% |  |  |
| GVHD – diagnosis and therapy | 74% | 11% | 6% |  |  |
| Pharmacology of conditioning and GVHD prophylaxis | 67% | 10% | 5% |  |  |

Abbreviations: SOT, solid organ transplant; HCT, hematopoietic cell transplant; GVHD, graft-versus-host disease; CTC, Comprehensive Trainee Curriculum

**Supplemental Figure 3: Fellows self-reported case volume by patient type in a dedicated TID clinic**


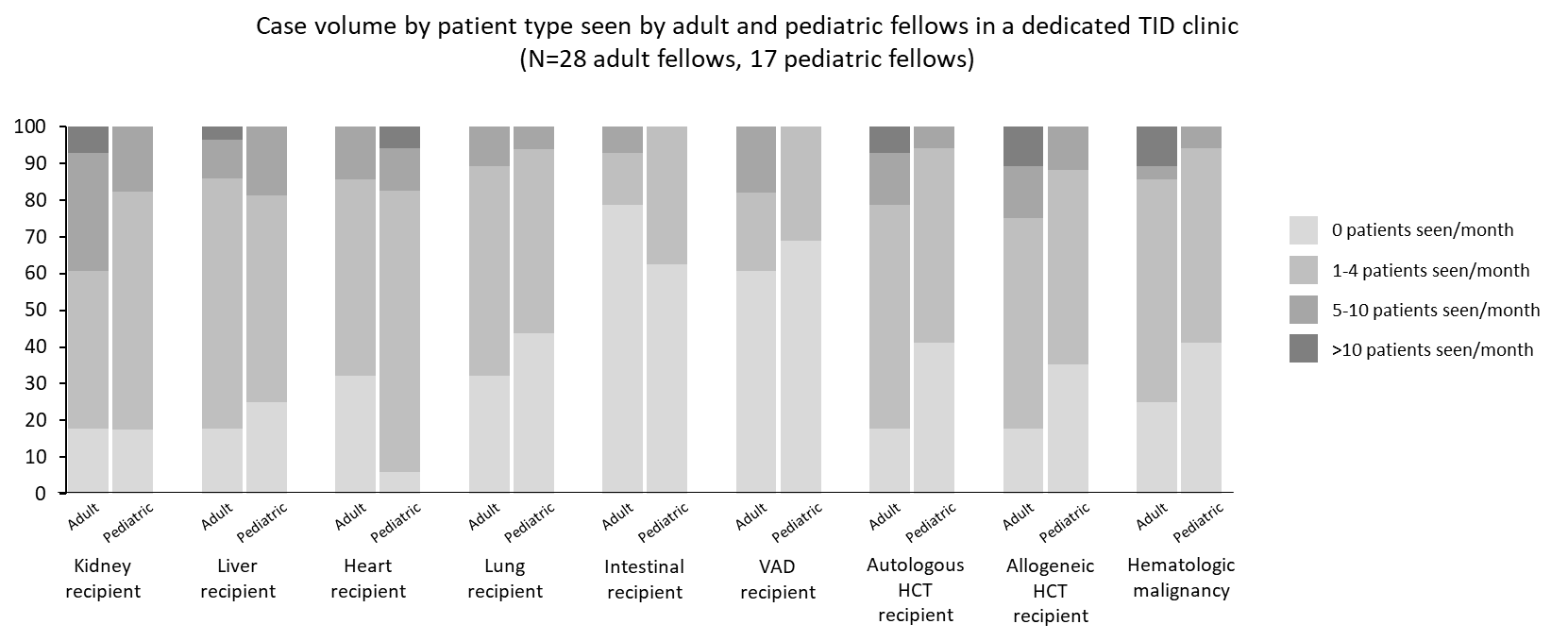


Abbreviations: HCT, hematopoietic cell transplant; SOT, solid organ transplant; TID, transplant infectious diseases; VAD, ventricular assist device

**Supplemental Figure 4: Fellow participation in non-clinical and professional TID development experiences**

Abbreviations: TID, Transplant Infectious Diseases; M&M, Morbidity and Mortality
